# Supplementary material for: The Evolution and Characterization of the RNA Interference Pathways in Lophotrochozoa
Source: Genome Biol Evol. 2024 May 7;16(5):evae098. doi: 10.1093/gbe/evae098 (PMC11114477; doi:10.1093/gbe/evae098)
Supplement: evae098_Supplementary_Data [file evae098_supplementary_data.zip › Supplementary_materials.zip!/selected_miRNAs/NoGe_selected_miRNAs/known_miRNA_families_pdf_files/NMRB01000012.1_Notospermus_geniculatus_strain_Ushimado_scaffold12,_whole_genome_shotgun_sequence_567.pdf]

Provisional ID	:	NMRB01000012.1_Notospermus_geniculatus_strain_Ushimado_scaffold12,_whole_genome_shotgun_sequence_567
Score total	:	5344.4
Score for star read(s)	:	3.9
Score for read counts	:	5336.5
Score for mfe	:	1.1
Score for randfold	:	0
Score for cons. seed	:	3
Total read count	:	10479
Mature read count	:	10398
Loop read count	:	0
Star read count	:	81

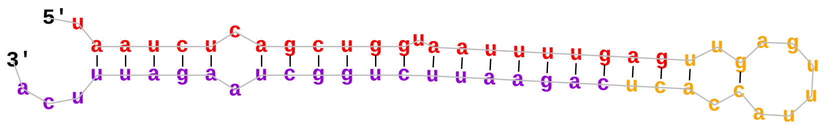

Genome coordinates: NMRB01000012.1 1032523-1032582

pre-miRNA sequence:
UAAUCUCAGCUGGUAAUUUUGAGUAAUCUCAGCUGGUAAUUUUGAGUUGAGUUUACCACUCAGAAUUCUGGCUAAGAUUUCA

Mature sequence: UAAUCUCAGCUGGUAAUUUUGAG

Aligned mature sequence of the *N. geniculatus* pre-miRNA with the top 5 hits from the BLAST search against the pre-miRNA mirGeneDB set:

```
                            ****************** ***
Nge_Mir-216-P2              uaaucucagcugguaauuuugag
Lan-Mir-216-P2e_5p          uaaucucagcugguaauuaugag
Lan-Mir-216-P2f_5p          uaaucucagcugguaauuaugag
Lgi-Mir-216-P2_5p           uaaucucagcugguaauucugag
Npo-Mir-216-P2k_5p          uaaucucagcugguaauuauga-
Cgi-Mir-216-P2_5p           uaaucucagcugguaauucugag
```
